# Aminosilane-Assisted Electrodeposition of Gold Nanodendrites and Their Catalytic Properties

**DOI:** 10.1038/srep39839

**Published:** 2017-01-03

**Authors:** Nga Yu Hau, Peixian Yang, Chang Liu, Jian Wang, Po-Heng Lee, Shien-Ping Feng

**Affiliations:** 1 Department of Mechanical Engineering, The University of Hong Kong, Pokfulam, Hong Kong; 2 Department of Civil and Environmental Engineering, The Hong Kong Polytechnic University, Hong Kong

## Abstract

A promising alternative route for the synthesis of three-dimensional Au dendrites was developed by direct electrodeposition from a solution of HAuCl_4_ containing 3-aminopropyltriethoxysilane (APTS). Ultraviolet-visible spectroscopy, fourier transform infrared spectroscopy and isothermal titration calorimetry were used to study the interaction of APTS in electrolyte. The effect of APTS on the formation of the hierarchical structure of Au dendrites was investigated by cyclic voltammetry, rotating disk electrode, electrochemical impedance spectroscopy and quartz crystal microbalance. The growth directions of the trunks and branches of the Au dendrites can be controlled by sweep-potential electrodeposition to obtain more regular structures. The efficacy of as-synthesised Au dendrites was demonstrated in the enhanced electro-catalytic activity to methanol electro-oxidation and the high sensitivity of glucose detection, which have potential applications in direct-methanol fuel cells and non-enzymatic electrochemical glucose biosensors, respectively.

Gold nanocrystals (NCs) have attracted a great deal of attention due to their nontoxic nature and unique size- and shape-dependent properties that have a wide variety of applications, including catalysis^1^,^2^, electronics^3^, sensing^4^, superhydrophobicity^5^, drug delivery^6^,^7^, bioimaging^8^ and photothermal therapy^9^,^10^. To date, hundreds of shape-controlled synthetic methods have been devised to produce Au NCs of various sizes and shapes^11^,^12^,^13^,^14^,^15^,^16^. Amongst them, great interest has been shown in hyper-branched dendritic structures^17^,^18^ because of their high surface-to-volume ratio and richness in active sites (e.g., tips, kinks, ledges, steps, and sharp edges) that lead to enhanced electro-catalytic activity. Most of the reported Au NCs are synthesised using templates^19^, organic additives or surfactants^20^,^21^, but the use of templates complicates the synthetic procedure, and the use of organic additives and surfactants may block active sites and introduce heterogeneous impurities. In addition, these synthetic methods, including uniform seed or template preparation, intermediate purification, adsorbate removal and template dissolution, are complicated and time-consuming.

Electrodeposition is a cost-effective approach to rapidly and directly grow Au dendrites in an aqueous solution. The electro-crystallisation can be readily manipulated by the interplay between the crystal growth rate and the mass transport rate via the control of deposition potential without changing the reactant concentration^22^,^23^,^24^. Ye *et al*.^25^ electroplated hierarchical Au dendrites in HuAuCl_4_ solution in the presence of Na_2_SO_4_ because the small ion of SO_4_^2-^ prefers to adsorb on Au [111] planes and block their growth. To improve the anisotropic growth rate to achieve dendritic structures with a high aspect ratio, the pulse potential was used in HuAuCl_4_ solution with the addition of cysteine as a capping agent^26^, which was removed selectively from [111] planes and remained on [110] and [100] planes during the potential-controlled process. However, the strong Au-S covalent bond may cause SH groups of cysteine to remain on the Au surfaces to hinder catalytic activity. Therefore, amine-group additives, such as ethylenediamine^12^, cytosine^27^ and L-asparagine^28^, were used as growth-directing agents in the electrodeposition of Au dendrites with ‘clean’ surfaces because they were selectively adsorbed onto specific Au surfaces by the moderate interactions. In reviewing the previous reported experiments, some challenges remain in this research area for electroplated Au dendrites. Methods of Au dendrite fabrication and their comparison were summarised in the Table S1 in the supporting information. First, electrodeposition usually takes hours to obtain Au dendrites with a high aspect ratio. Second, this lengthy electroplating process readily causes precipitation in HuAuCl_4_ solution, particularly when capping agents with strong binding to Au^+^ ions are used. Finally, the objective is to produce Au dendrites with more regular structures for reproducible and predictable catalytic performance.

In this study, effective electrodeposition of high aspect-ratio Au dendrites was developed by using 3-aminopropyltriethoxysilane (APTS) as an additive in HAuCl_4_ solution. An addition of low concentration (0.1 vol%) of APTS in the electrolyte is used to electroplate Au dendrite; the electrolyte is very stable and can be stored for months, which is suitable for mass production. The entire electroplating process was completed within 10 min in a stable electrolyte. To date, the control of growth direction is still a challenge in the electrodeposition of nanostructures. In this work, the growth directions of the trunks and branches of the Au dendrites can be controlled by sweep-potential to obtain more regular structures. We demonstrated the efficacy of as-synthesised Au dendrites in the enhanced electro-catalytic activity to methanol electro-oxidation and the high sensitivity of glucose detection.

## Results and Discussion

### Role of APTS in gold dendrite electrodeposition

**Figure 1:**
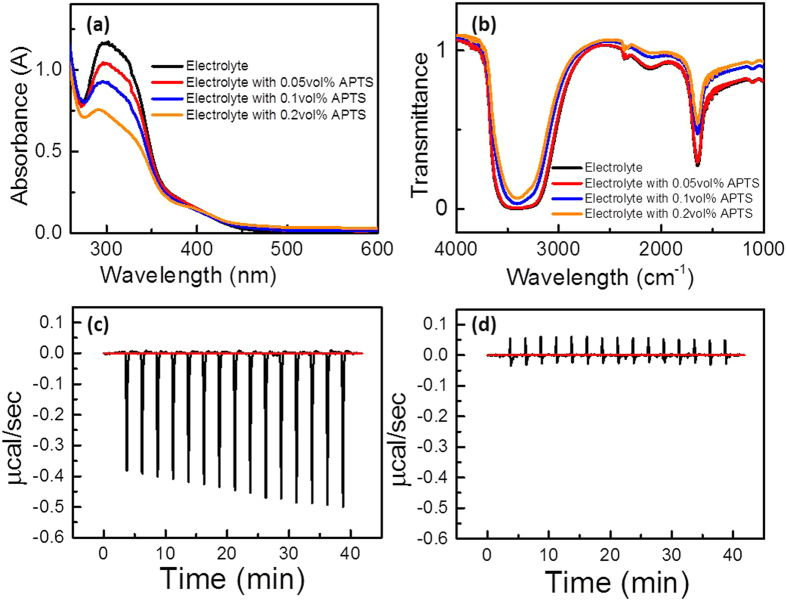
(**a**) UV-visible spectrum of gold electrolyte with addition of different concentrations of APTS. (**b**) FTIR spectrum of gold electrolyte with addition of different concentrations of APTS. (**c**) ITC titration data describing interaction between APTS and precursor with 50 μM HAuCl_4_ and 50 μM HClO_4_. (**d**) ITC titration data describing interaction between APTS and 50 μM HClO_4_. 5 μL of 750 mM APTS was injected in 200 μL of stock solution in each injection.

**Figure 2:**
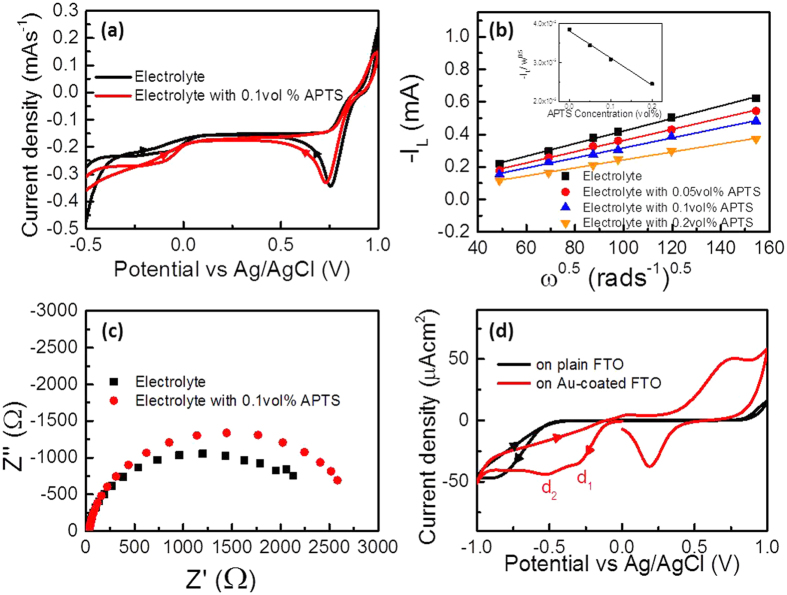
(**a**) Cyclic voltammogram of gold electrolyte with and without APTS on FTO glass. (**b**) Plot of –I_L_ against *ω*^0.5^ of different concentrations of APTS in gold electrolyte at platinum rotary disc electrode. (inset: plot of slope of –I_L_ against *ω*^0.5^ vs concentration of APTS) (**c**) Nyquist plot of EIS measurement of gold electrolyte with and without APTS on FTO glass. (**d**) Cyclic voltammogram of 0.1% APTS in DI water on plain FTO and gold electrode.

The ultraviolet-visible spectrum in Fig. 1(a) shows the absorption peak corresponding to AuCl_4_^−^ at wavelength of 311 to 314 nm^29^ decreases as the concentration of APTS increases, which indicates the formation of complex in the electrolyte. The Fourier transform infrared spectrum in Fig. 1(b) also provides evidence that the wavelength of 1500 to 1700 nm decreases as the concentration of APTS in the electrolyte increases, which is mainly associated with C=O stretching vibration and N–H bending vibration^30^. Isothermal titration calorimetry^31^ was used to understand the binding interaction between AuCl_4_^−^ and APTS by measuring the small energy change (μW) when APTS is added into the electrolyte. Each negative peak shown in the heat signal curves in Fig. 1(c) represents an exothermic process, which denotes the heat released in one injection of APTS into the electrolyte containing 50 μM HAuCl_4_ and 50 μM HClO_4_ as a function of time. For comparison, Fig. 1(d) is the plot of the heat response against time when APTS is added into the reaction vessel with the electrolyte only containing 50 μM HClO_4_, which shows no apparent negative or positive peaks. This result indicates that the formation of complex is mainly between Au ions and APTS. The cyclic voltammetry curves in Fig. 2(a) show that the cathodic curve of Au reduction shifts to more negative potential in the presence of APTS in the electrolyte because the complex increases the formation energy in the early stage of electroplating^32^. Therefore, the reduction in the Au-APTS complex requires higher potential than that of Au ions in electrolyte. A rotating disk electrode was used to investigate the electrolyte without and with different concentrations of APTS at 1000 rpm, as shown in Fig. 2(b). According to the Levich equation^33^: 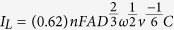, where *I*_*L*_ is the limiting current, n is the number of involved electron, *F* is the Faraday’s constant, *A* is the surface area of the platinum disc electrode (0.283 cm^2^), *D* is the diffusion coefficient, *ω* is the angular rotation rate of the electrode, *v* is the kinematic velocity and C is the bulk concentration of the redox pair ions. Therefore, the slope of the graph *I*_*L*_ vs 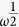 is proportional to the diffusion coefficient of the system, which decreases as the concentration of APTS increases. The formation of Au-APTS complex causes the diffusion coefficient to decrease, which plays an important role in the formation of dendritic Au nanostructures due to the diffusion-limited growth process^33^,^34^.

### Potential-controlled electrodeposition of gold dendrites

**Figure 3:**
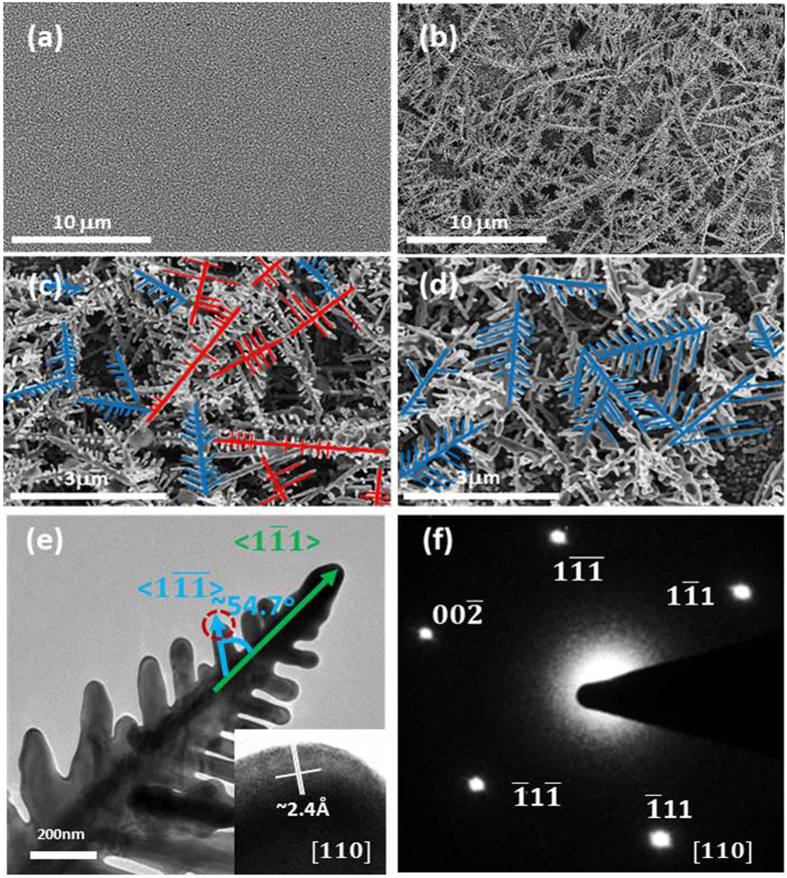
SEM and TEM images of gold nanoparticles and gold dendrites. SEM images of deposited gold crystals under −0.25 V for 300 s on FTO-glass (**a**) without and (**b**) with 0.1% APTS addition in electrolyte. SEM images of deposited gold crystals under (**c**) constant voltage −0.25 V for 300 s and (**d**) sweeping potential −0.5 V to 0 V with 0.1% APTS addition in electrolyte on FTO-glass. (**e**) TEM images of Au dendrite obtained by sweeping potential −0.5 V to 0 V with 0.1% APTS addition in electrolyte on FTO-glass. (Inset: (**f**) SAED pattern and (**g**) HRTEM image of the corresponding TEM image).

Similar to the bond formation between thiol molecules and gold, which is more common in use, the amino-group of APTS can be bonded moderately^35^ on the gold nuclei surface via the lone pair of nitrogen^36^ during the initial electroplating nucleation process. This causes the –NH_2_ group to be held parallel to the surface plane while the tail group (−OC_2_H_5_) is tilted with respect to the surface normal^2^,^37^. This hence forms a bulky sheath to increase the interfacial resistance between the surface during electrodeposition and the solution to induce anisotropic growth of Au crystal. Electrochemical impedance spectroscopy was used to characterise the interfacial behaviour in the electrolyte with and without APTS, as shown in Fig. 2(c). The Au-coated fluoride–tin oxide (FTO) used as a working electrode was immersed in the solution for 30 s, followed by electrochemical impedance spectroscopic measurement in the open circuit condition. The increase in charge transfer resistance for the case with APTS provides evidence that the APTS would adsorb on the Au surface via electrostatic attraction, resulting in an increase in interfacial resistance. The previous study reported that the binding of APTS on Au [110] and [100] is stronger than that on Au [111] because the relative surface energies of the crystallographic planes are [110] > [100] > [111] in face-centred cubic (fcc) Au metal^11^,^15^. Reductive desorption^38^,^39^ of APTS occurs on a negatively charged working electrode because the bond between the amino-group and gold originates with the nitrogen lone pair electrons. To investigate APTS desorption from the Au surface, distilled (DI) water was used to replace the electrolyte to prevent the continuity of Au electrodeposition in the system. Figure 2(d) shows cathodic waves for bare and Au-coated FTO in DI water with 0.1 vol% APTS, providing evidence that the electro-desorption of APTS from the Au surface occurs at a certain potential. The APTS adsorbs on the Au surface via electrostatic force, and the increased cathodic potential induces APTS desorption from the Au surface. For the Au-coated sample, the first peak (d_1_) at −0.25 V should correspond to APTS desorption on Au [111], and a more negative potential at −0.55 V (d_2_) corresponds to APTS desorption on Au [110] and [100]. As noted, the adsorption of APTS is related to bond via nitrogen lone pair^36^, whilst the desorption of APTS is a potential-dependent process^38^,^40^. Figure 3(a,b) show scanning electron microscopic (SEM) images of electroplating Au in electrolyte with and without 0.1 vol% APTS at the potential of −0.25 V for 300 s. The spherical Au nanoparticles were electroplated on FTO in the electrolyte whilst the forest of Au dendrites was obtained in the electrolyte with the addition of APTS. As shown in the SEM images in Fig. 3(c), the Au dendrites were prepared by constant potential electrodeposition, which comprised two groups of growth angles between the branches and the trunk in the same sample. As mentioned above, the desorption of APTS is potential-dependent based on the crystallographic planes of Au; the desorption of APTS occurs on Au [111] when the potential reaches −0.25 V or a more negative value. Therefore, during electrodeposition at −0.25 V, the exposed [111] leads to combination of the trunk and branches of an Au dendrite into a single crystal preferentially grown along the <111> direction^26^, while the angles are nearly the theoretical angle of 54.7° between two <111> directions of fcc structure under the projection view of [110]. The other type of Au dendrites with perpendicular angles between the branches and the main trunk also existed in the same sample. Given a constant operating potential, the electron accumulation causes a range of potential distribution throughout the formed Au clusters on the electrode, which may induce local APTS desorption^26^ on Au [110] and [100], resulting in branches grown along <110> direction taking perpendicular angles. To improve this, the sweeping potential with a scanning rate of 1 mV/s from −0.5 V to 0 V was introduced to compensate for the potential distribution to control the growth directions of the trunks and branches of the Au dendrites. When the potential is initially swept at −0.5 V, the APTS was preferentially desorbed from Au [111]. The positive sweeping of potential can then effectively keep the APTS capping on [110] and [100], leading the Au dendrites grown along <111> to obtain more regular structures, as shown in the SEM images in Fig. 3(d). One of the Au dendrites was examined by transmission electron microscopy and selective area electron diffraction, as shown in Fig. 3(e). In the projection view of [110], the angle between two <111> directions is approximately 54.7°. The [111] high-resolution transmission electron microscopic image of the Au dendrite in the inset of Fig. 3(e) shows a single crystal Au, and the measured lattice fringes with d spacing of 2.4 Å is equal to the [111] lattice spacing of the fcc Au crystal, which is consistent with the diffraction pattern given in Fig. 3(f). The corresponding selected area diffraction patterns obtained from the zone axis [110] exhibited four strong spots in two-fold symmetry, corresponding to the [111] reflections (d-spacing, 2.4 Å) of the fcc Au single crystal.

XRD patterns of Au NPs obtained from single potential deposition without APTS, Au dendrites obtained from single potential deposition with APTS and Au dendrites obtained from sweeping potential deposition with APTS are shown in Figure S1(a) in the supporting information. All prepared Au samples show typical XRD pattern with the peaks at 39.8^o^, 46.4^o^, 64.6^o^, 77.6^o^, which are assigned to [111], [200], [220], [311] of Au fcc crystal diffraction respectively (JCPDF No.04-0802). The Au dendrites prepared by sweeping potential deposition with APTS has more [111] facets resulting in a higher [111]/[200] ratio as compared to other samples, as shown in Figure S1(b–e) in the supporting information (normalized with respect to the [111] peak)^25^.

### Electro-oxidation of methanol

**Figure 4:**
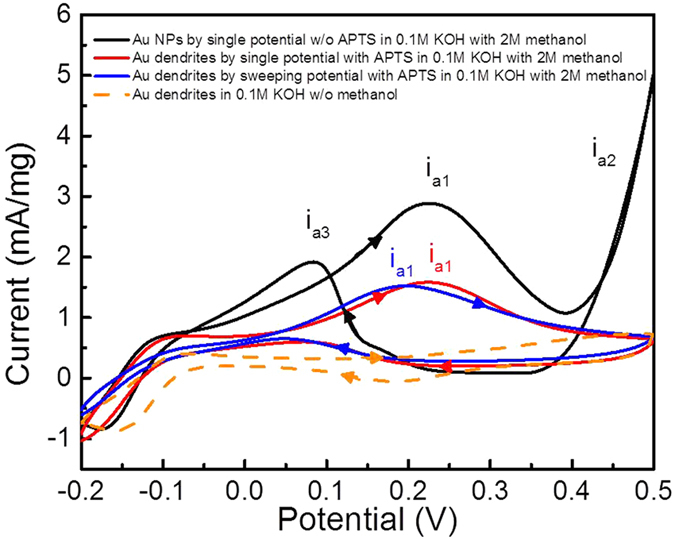
Electro-oxidation of methanol. Cyclic voltammograms for Au NPs obtained from single potential deposition without APTS, Au dendrites obtained from single potential deposition with APTS and Au dendrites obtained from sweeping potential deposition with APTS in 0.1 M KOH with and without addition of 2 M methanol.

The synthesised Au dendrites were used to demonstrate their catalytic ability in the oxidation of methanol and glucose. The oxidation of methanol to carbon dioxide is important for direct-methanol fuel cells; the anodic reaction is as follows^41^,^42^,^43^: 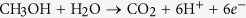 in this reaction, the Au nanostructure can serve as a good catalyst because Au [111] allows effective cleavage of the O-H bond to form surface bond methoxy^2^,^44^. In Fig. 4, no significant cathodic peak can be found in the orange dashed curve that represents the cyclic voltammogram of the Au dendrite electrode (prepared by sweeping potential deposition in APTS-containing electrolyte) in 0.1 M KOH without methanol. However, three cathodic peaks were recorded in the black curve corresponding to the current response of the Au nanoparticles, (prepared by single potential deposition without APTS) in 0.1 M KOH with 2 M methanol. In the forward scan, the formation of a cathodic peak i_a1_ at 0.225 V was contributed by the oxidation of methanol with the reaction equations below^42^,^45^: 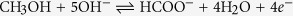. Under the same operating conditions, Au dendrite electrodes obtained by single and sweeping potential in APTS-containing electrolyte have the anodic peaks i_a1_ at 0.225 V and 0.192 V respectively. It indicates that the [111] facet dominated Au dendrite electrode deposited by sweeping potential with APTS has a lower reaction barrier for methanol oxidation. This is also a relatively low anodic peak voltage as compare with the previous reported Au nanostructures for methanol oxidation, as shown in Table S2. At higher potential, methanol molecules are oxidised to form carbonates and the cathodic peak i_a2_ starts at 0.42 V according to the reaction as follows^42^,^46^: 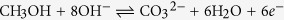. The carbonate intermediates from this oxidation were further oxidised^44^ in the backward scan and caused the cathodic peak i_a3_ at 0.08 V. The transfer process of carbonates recognised in peak i_a2_ and i_a3_ is related to the poisoning effect of the oxidative catalyst, which is usually not desirable for methanol oxidation^47^,^48^,^49^. As seen in the blue curve in Fig. 4, only one cathodic peak of i_a1_ can be seen in both forward and backward scans for the dendritic Au electrode, which indicates that reproducible and predictable catalytic performance can be produced with the use of Au dendrites with regular structures.

### Glucose detection

**Figure 5:**
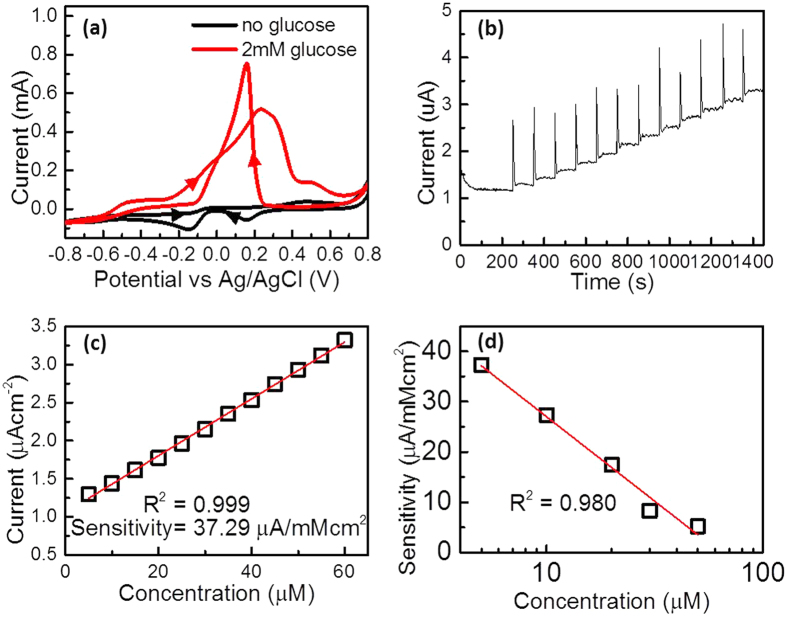
Oxidation of glucose. (**a**) Cyclic voltammograms for 0.1 M NaOH at the [111] facet dominated Au dendrite electrode deposited by sweeping potential with APTS with and without 2 mM glucose. (**b**) Amperometric response of gold dendritic electrode under successive addition of 5 μM of glucose at 0.3 V. (**c**) Linear calibration and (**d**) sensitivity as a function of glucose concentrations.

Enzyme-based biosensors have been used to detect blood glucose in patients with diabetes on the basis of glucose oxidase bound to enzyme electrode. However, their detection limit is not effective for the detection of glucose levels in other sources, such as tears and saliva (which are 38 to 500 times lower than the concentration in blood glucose)^50^. In this study, the [111] facet dominated Au dendrite electrode deposited by sweeping potential with APTS were used as a sensitive electrode for potential application in a non-enzymatic electrochemical glucose biosensor. As shown in the cyclic voltammetry curves in Fig. 5(a), in the absence of glucose, the cathodic peaks of the reverse scan at 0.16 V and 0.14 V correspond to the reduction of gold oxide produced in the forward scan. In the presence of glucose, the anodic peaks in the forward scan at −0.46 V correspond to the peaks of partial oxidation of glucose, which causes electrosorption of glucose to form adsorbed intermediate, and the anodic peak at 0.24 V represents the direct oxidation of glucose to gluconolactone^51^,^52^. The reverse scan shows a strong anodic peak at 0.16 V that is formed by the direct oxidation of glucose. Because the AuOH on the gold crystal surface is the active site for electro-catalytic oxidation of glucose to take place, the population of AuOH sites is greatly enhanced in the forward scan^51^,^52^,^53^; hence, direct oxidation of glucose occurs in the reverse scan. To determine the sensitivity of the dendritic Au as a glucose biosensor, the amperometric responses of the electrode were recorded with the successive addition of different concentrations of glucose at 0.3 V. By calculating the slope of the calibration of the amperometric response, the sensitivity of the dendritic electrode under certain concentration can be evaluated^54^,^55^. The amperometric response and linear calibration of the response of the concentration of 5 μM of glucose are shown in Fig. 5(b,c), while those of concentrations of 10, 15, 30 and 50 μM are shown in Figure S2 in the supporting information. The sensitivity of the dendritic electrode in different concentrations of glucose is summarised in Table 1. The dendritic Au electrode can achieve a relatively low detection limit of 5 μM with a sensitivity of 37.29 μA/cm^2^·mM. Another important feature that makes the dendritic Au electrode a good candidate for a non-enzymatic glucose sensor is its wide linear range in sensitivity against glucose concentrations from 5 to 50 μM, as shown in Fig. 5(d). This result is expected to pave the way for non-enzymatic electrochemical glucose biosensors for non-invasive glucose testing of tears and saliva^56^,^57^.

## Discussion

**Figure 6:**
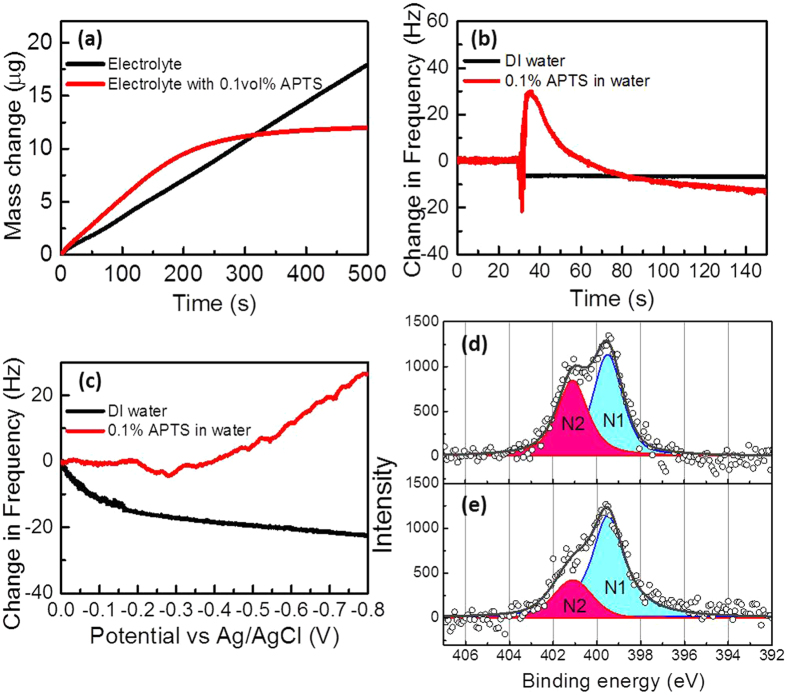
Mass change and APTS adsorption/desorption on gold QCM electrode and XPS spectrum. (**a**) Mass change on QCM electrode vs time in gold electrolyte with and without APTS addition under sweeping potential from −0.5 V to 0 V at scan rate of 1 mVs^−1^. (**b**) Change in vibration frequency of QCM electrode in 2 mL DI water before and after injection of DI water and 20 μL of 10% APTS. (**c**) Change in vibration frequency of QCM electrode in DI water and 0.1% APTS in water under sweeping potential from 0 V to −0.8 V at 10 mVs^−1^ scanning rate. N1s core level XPS measurement and fitting obtained from Au-coated silicon wafer (pre-soaked in 10%APTS for 1 minute) (**d**) before and (**e**) after applying the sweeping potential from 0 V to −0.5 V at scan rate of 10mVs^−1^ in DI water.

Quartz crystal microbalancing (QCM) was carried out to understand the mass change of electroplating Au in the electrolyte with and without APTS with the use of the sweeping potential waveform. With the piezoelectric property of quartz crystal, the change in mass can be calculated from the measured change in the electrode’s oscillation frequency with the Sauerbrey equation^58^. As shown in Fig. 6(a), the normal electrodeposition of Au on a foreign substrate usually follows Volmer-Weber growth^59^, in which the nucleation sites grow into islands and then coalesce as a thin film (black curve). With the addition of APTS (red curve), the occurrence of plateaus indicates that the growth of the electroplated Au dendrites stopped after 200 s because the increased electrical resistance raised a formation barrier of electrodeposition when the branches of the dendritic structure elongated to certain lengths. In Fig. 6(b), the resonant frequency of the Au quartz crystal electrode was measured in a Teflon cell with 2 mL DI water by adding 10 vol% APTS. After adding APTS to the DI water at 30 s, the change in the frequency became positive in the beginning due to the inference of the inject droplet momentum and then became negative (red curve). The negative change in the frequency resulted from the increased weight of the electrode, suggesting that APTS was adsorbed on the Au quartz crystal electrode via electrostatic force. In comparison with the addition of DI water, the change in frequency fluctuated in the beginning and then remained stable (black curve). Furthermore, QCM was performed with a sweeping potential from 0 V to −0.8 V at a scanning rate of 1 mVs^−1^ with 2 mL of DI water and DI water with 0.1 vol% APTS. The change in the resonant frequency against the applied potential is shown in Fig. 6(c). The change in frequency was not obvious at the potential before −0.2 V in the DI water with 0.1% APTS and then gradually became positive after the potential over −0.25 V. The positive change means that the weight of the electrode decreases, which suggests that APTS desorption occurs when the potential is over −0.25 V, which is consistent with the mechanism mentioned above. In contrast, the weight of the electrode was increased in DI water under the sweeping potential due to the formation of gas bubbles^60^, resulting in the negative change in the frequency.

Au-coated silicon wafers, which were pre-soaked into 10% APTS solution for 1 minute, were used to measure XPS before and after applying sweeping potential from 0 V to −0.5 V at scan rate of 10mVs^−1^ in DI water. To understand the weak covalent binding of amine to Au^35^,^61^, the analysis of N1s core level are shown in Fig. 6(d,e). Both spectra have board and asymmetry feature in the binding energy in the range of 396–403 eV, showing one neutral amino group (NH_2_) marked as N1 at 399.5 eV and the other positive amine nitrogen (NH_3_^+^) marked as N2 at 401.1 eV^62^,^63^. As Au and amine has a stronger bond in +1 charge state than in neutral state^64^, N2 can represent the interaction between Au and nitrogen, which is decreased after applying sweeping potential. This agrees with the results of QCM and ITC, providing evidence that APTS is bonded to Au surface and the APTS desorption occurs when applying a negative potential. N1s core level analysis was also done on the Au NPs obtained by single potential without APTS and Au dendrites obtained by sweeping potential with APTS. As shown in Figure S3 in the supporting information, the intensity of the peak assigned to N1s core level obtained from Au dendrite is higher than that obtained from the Au NPs, which provides evidence of the ATPS adsorption on the Au dendrites.

In conclusion, a promising alternative electroplating route was developed to synthesise hyper-branched Au dendrites with the aid of APTS as a growth-directing agent without the use of seed, surfactant or template. This approach is based on the electrostatic absorption of APTS and selective electro-desorption of APTS from crystallographic planes of Au under a controlled potential. The sweeping potential technique can be used to effectively maintain APTS capping on [110] and [100], and thus crystalline Au preferentially grew along the <111> directions to form structures with more regular angles between their trunks and branches. The as-prepared Au dendrites exhibited enhanced electro-catalytic activity to electro-oxidation of methanol for possible application in direct-methanol fuel cells. The Au dendrites also showed a low detection limit of 5 μM with a wide linear range from 5 to 50 μM for non-enzymatic glucose detection. This strategy of selective desorption with an appropriate growth agent using a suitable sweeping potential technique might be applicable to the electrodeposition of other metal or bimetallic nanostructures.

## Methods

### Fabrication of Au dendrites

The FTO glass (2.2 mm thick, sheet resistance 10 Ω/□) substrate was cleaned in glass cleaner for 20 min and then in DI water for another 20 min under ultrasonic process. An electrochemical workstation (CHI 660E) was applied to a standard three-electrode system with the clean FTO as the working electrode, platinum mesh as the counter electrode and saturated Ag/AgCl as the reference electrode. The electrolyte was composed of 3.4 mM HAuCl_4_, 0.1 M HClO_4_ and different amounts of APTS at 0.05, 0.1 and 0.2 vol%. Constant potential electrodeposition was used to electroplate Au on the FTO surface at −0.25 V for 300 s. The sweeping potential electrodeposition was started from −0.5 V to 0 V at a scanning rate of 1 mVs^−1^. All samples were immersed in the electrolyte for 30 s before the electroplating process began.

### Electrochemical and Material Characterisation

A Fourier transform infrared spectrometer (Bruker Tensor 27) with liquid holder (CaF_2_ windows) and ultraviolet-visible spectrometer (Perkin Elmer Lambda 35) were used to evaluate the transmittance spectra for the electrolyte without and with different concentrations of APTS. Isothermal titration calorimetry (Malvern MicroCal™ iTC200) was used to investigate the interaction between the APTS and the electrolyte by injecting 5 μL of APTS (750 mM) into 200 μL of electrolyte (50 μM HAuCl_4_, 50 μM HClO_4_). The binding of APTS, HAuCl_4_ and HClO_4_ can be determined by the exothermic heat released. The concentration of APTS was elevated to ensure detectable signals. A standard three-electrode configuration consisting of FTO or Au-coated FTO as the working electrode, saturated Ag/AgCl as the reference electrode, and platinum mesh as the counter electrode was used in the electrolyte with or without APTS for cyclic voltammetry and electrochemical impedance spectroscopy. Quartz crystal microbalance (QCM, CHI430B, CH Instruments) was measured in a small Teflon cell of about 2 mL of electrolyte with and without APTS using Au-coated quartz crystal (active area, 0.196 cm^2^; resonant frequency of crystal in air, 7.995 MHz) as the working electrode, a platinum wire counter electrode and an Ag/AgCl reference electrode. The limiting currents of the electrolytes with different concentrations of APTS were measured with a rotating disk electrode (RDE PTE Platinum Disk Electrode 600C) at room temperature to determine the diffusion coefficients. The atomic structure, morphology and crystallinity of the Au dendrites were determined by high-resolution transmission electron microscopy (Tecnai, G220S-Twin) and field-emission SEM (Hitachi S-4800). X-ray diffraction (Bruker D8 Advance) was carried out to confirm the crystallinility of Au electrodes. X-ray photoelectron spectroscopy (XPS, Physical Electronics 5600) was applied to analyze the surface chemical compositions and the spectra were corrected with C1s core level as reference.

### Electro-oxidation of methanol and glucose detection

Two Au-coated FTO samples, Au nanoparticles and dendritic Au samples, were fabricated by electroplating Au on FTO in the electrolyte without and with 0.1 vol% APTS, respectively. The samples were placed in 0.1 M KOH solution with 2 M of methanol when cyclic voltammetry was applied from 0.5 V to −0.2 V at a scanning rate of 10 mVs^−1^. The ratios of the current peaks of the forward and backward scans were recorded to evaluate the catalytic performance for the electro-oxidation of methanol. For glucose detection, the Au-coated FTO was used as the working electrode and its current density was recorded under −0.3 V with the subsequent injection of 14 μL of glucose solution into a 10-mL bulk solution of 0.1 M NaOH. Different concentrations of glucose solution were applied to calibrate the sensitivity curve against the concentration of glucose.

## Additional Information

**How to cite this article**: Hau, N. Y. *et al*. Aminosilane-Assisted Electrodeposition of Gold Nanodendrites and Their Catalytic Properties. *Sci. Rep.* **7**, 39839; doi: 10.1038/srep39839 (2017).

**Publisher's note:** Springer Nature remains neutral with regard to jurisdictional claims in published maps and institutional affiliations.

## Supplementary Material

Supporting Information

## Figures and Tables

**Table 1:** Sensitivity of gold dendritic electrodes in 0.1 M NaOH with different concentrations of glucose.

Concentration	μM	5	10	15	30	50
Sensitivity	μA/mMcm^2^	37.29	27.31	12.44	7.90	5.2
