# Supplementary material for: Aminosilane-Assisted Electrodeposition of Gold Nanodendrites and Their Catalytic Properties
Source: Sci Rep. 2017 Jan 3;7:39839. doi: 10.1038/srep39839 (PMC5206629; doi:10.1038/srep39839)
Supplement: Supporting Information [file srep39839-s1.pdf]

Aminosilane-Assisted Electrodeposition of Gold Nanodendrites and Their Catalytic Properties

Nga Yu Hau[1], Peixian Yang[2], Chang Liu[1], Jian Wang[2], Po-Heng Lee[2], Shien-Ping Feng[1*]

[1]Department of Mechanical Engineering, The University of Hong Kong, Pokfulam, Hong Kong

[2]Department of Civil and Environmental Engineering, The Hong Kong Polytechnic University, Hong Kong

Supporting Information:

Table S1 Summary of fabrication methods of Au dendrites and description

Fabrication method	Description	Ref
**Ionic-liquid assisted deposition of gold dendrite with amino groups additives**	Reacts on zinc plate at 60°C for 4.5 hours. The product consisted of irregular dendrites and many unusual four-branched dendrites.	1
**Gold dendrite by electrolysis of molten chloride**	500-700°C of high temperature needed. The dendrites are the major form of gold deposits.	2
**Gold dendrimer by reduction**	Ionic polymer template needed. Takes at least 10 days for crystal growth in irregular dendrimer form.	3
**Gold dendrimer by nanoparticle self-assembly**	Surfactant needed. Takes at least 3 days for crystal growth in irregular dendrimer form.	4
**This work**	No templates and surfactants. Stable electrolyte which can last for months. Rapid deposition within 10 min. Regular dendrite structure are obtained.	

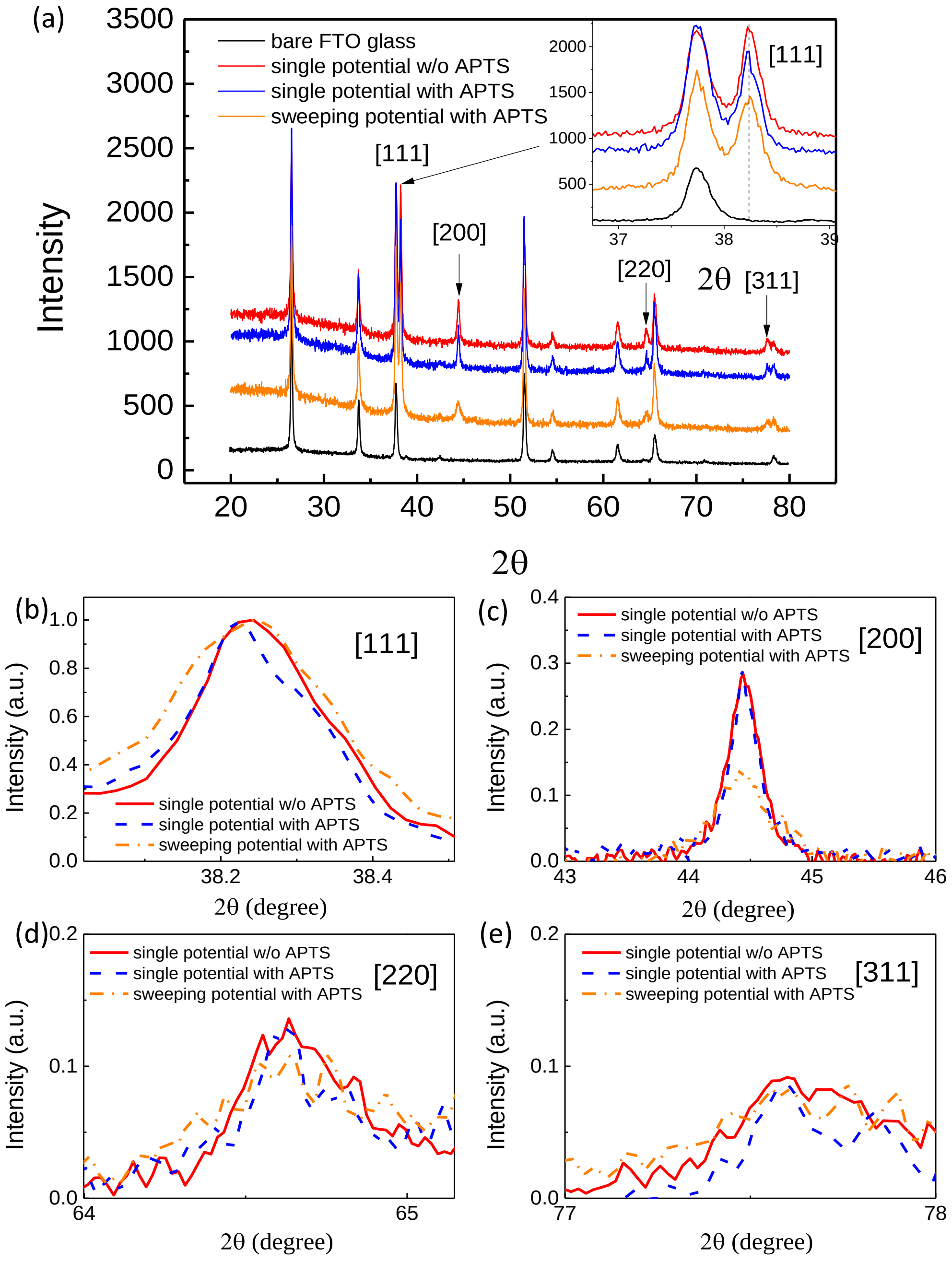

Figure S1 (a) XRD patterns of Au NPs obtained from single potential deposition without APTS, Au dendrites obtained from single potential deposition with APTS and Au dendrites obtained from sweeping potential deposition with APTS and peaks of (b) [111], (c) [200], (d) [220] and (e) [311] facet of normalized with respect to the [111] peak.

Table S2: Au nanostructures and its catalytic performance in oxidation of methanol

	**Anodic peak of glucose oxidation in alkaline medium (V)**	**Ref**
**Carbon black supported Au nanoparticle**	0.357	5
**Nanoporous gold**	0.44	6
**Au-Pt alloy nanoparticle**	0.3	7
**Self-supported nanoporous Au film**	0.27	8
**Core-shell assembled gold nanoparticles**	0.215	9
**This work**	0.192	

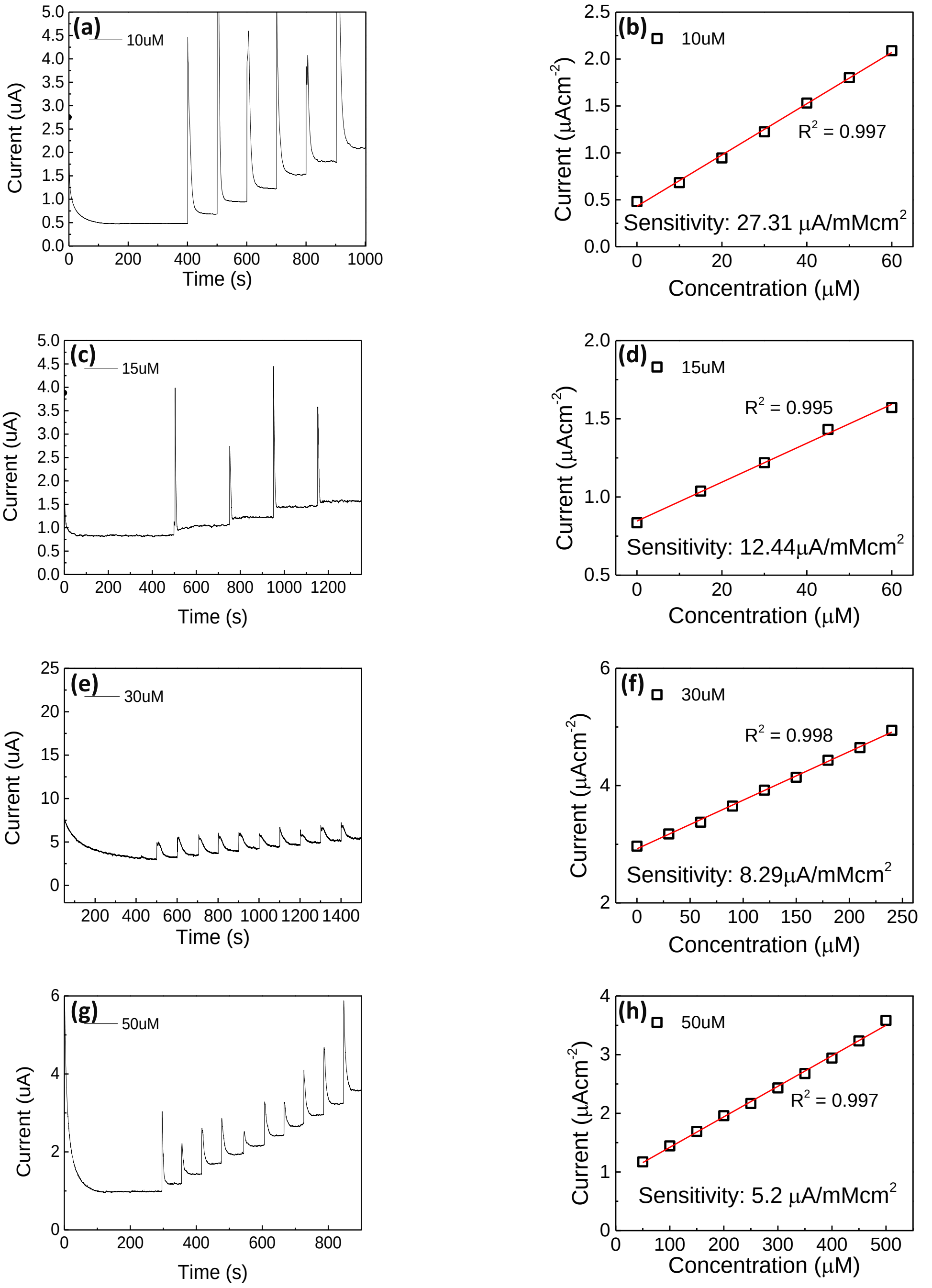

Figure S2. Amperometric response of gold dendritic electrode under successive addition of (a) 10 µM, (c) 15 µM, (e) 30 µM and (g) 50µM of glucose at 0.3 V and their corresponding linear calibration (b) 10 µM, (d) 15 µM, (f) 30 µM and (h) 50 µM of glucose.

Table S3: Au nanostructures and its catalytic performance in oxidation of glucose

	**Detection limit (μM)**	**Sensitivity (μAmM^{-1}cm^{-2})**	**Ref**
**Au nanowire array**	20000	309.0	10
**Gold nanoparticles-mesoporous silica composite**	15	6.1	4
**Au nanoparticle-constituted nanotube array**	10	1.13	5
**Multilayer films composed of multi-wall carbon nanotubes, gold nanoparticles and glucose oxidase**	6.7	2.527	2
**Au nanoparticles/multiwalled carbon nanotubes/glucose oxidase membrane**	128	7.3	7
**This work**	5	37.29	

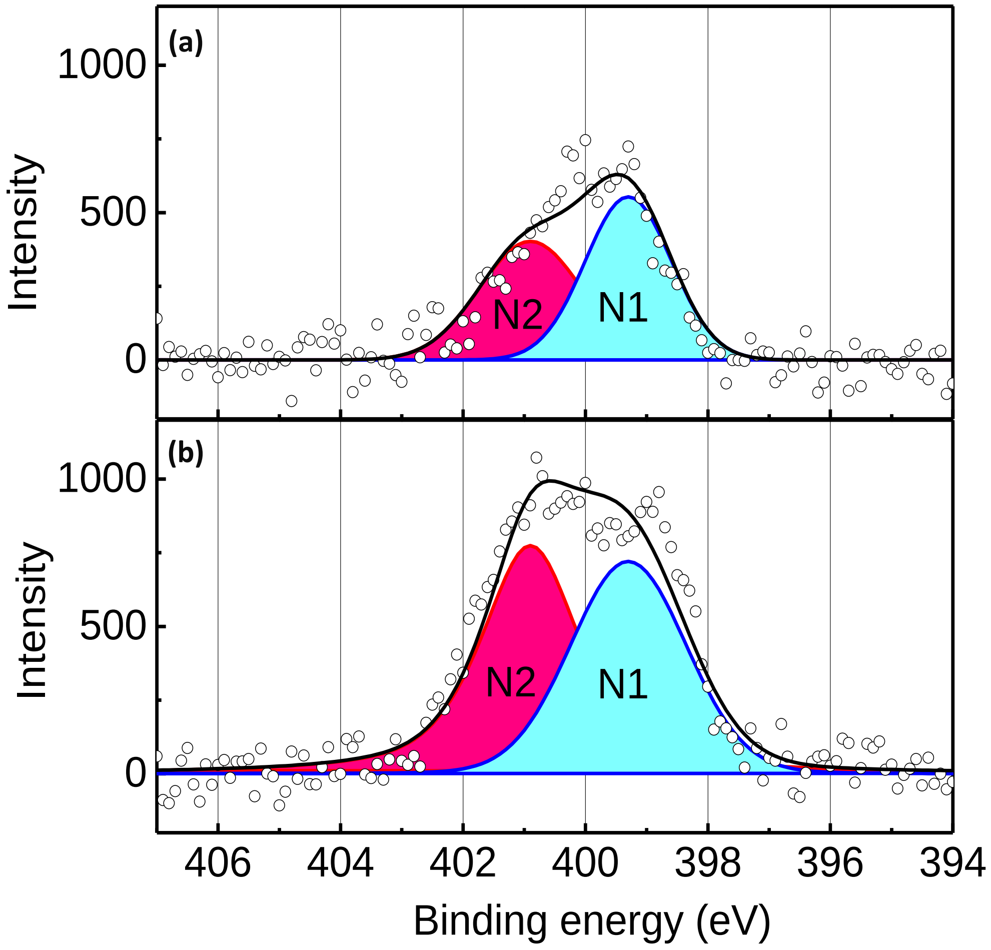

Figure S3 N1s core level XPS measurement and fitting obtained from (a) Au NPs deposited by single potential deposition without APTS and (b) Au dendrites deposited by sweeping potential deposition with APTS. (normalization with respect to the peak of Au4f)

References:

1 Qin, Y. *et al.* Ionic Liquid-Assisted Growth of Single-Crystalline Dendritic Gold Nanostructures with a Three-Fold Symmetry. *Chem. Mater.* **20**, 3965-3972 (2008).

2 Saltykova, N. A., Semerikova, O. L. & Molchanova, N. G. Dendrite structure of electrolytic gold deposited from molten chlorides. *Russ. J Electrochem.* **43**, 863–869 (2007).

3 Zhang, J. *et al.* Fabrication of Dendritic Gold Nanoparticles by Use of an Ionic Polymer Template. *Langmuir* **24**, 2699–2704 (2008).

4 S. Pang , T. K., T. Kawai Formation of Dendrimer-like Gold Nanoparticle Assemblies. *Chem. Mater.* **17**, 3636–3641 (2007).

5 Luo, J. *et al.* Electrocatalytic oxidation of methanol: carbon-supported gold–platinum nanoparticle catalysts prepared by two-phase protocol. *Catal. Today* **99**, 291-297 (2005).

6 Zhang, J., Liu, P., Ma, H. & Ding, Y. Nanostructured Porous Gold for Methanol Electro-Oxidation. *J. Phys. Chem. C* **111**, 10382–10388 (2007).

7 Lou, Y., Maye, M. M., Han, L., Luo, J. & Zhong, C.-J. Gold–platinum alloy nanoparticle assembly as catalyst for methanol electrooxidation. *Chem. Commun.*, 473-474 (2001).

8 Yu, C., Jia, F., Ai, Z. & Zhang, L. Direct Oxidation of Methanol on Self-Supported Nanoporous Gold Film Electrodes with High Catalytic Activity and Stability. *Chem. Mater.* **19**, 6065–6067 (2007).

9 Luo, J. *et al.* Catalytic activation of core-shell assembled gold nanoparticles as catalyst for methanol electrooxidation. *Catal. Today* **77**, 127–138 (2002).

10 Cherevko, S. & Chung, C.-H. Gold nanowire array electrode for non-enzymatic voltammetric and amperometric glucose detection. *Sens. Actuators B Chem.* **142**, 216-223 (2009).
